# Cohort profile: The Japan Environment and Children’s Study

**DOI:** 10.1093/ije/dyag146

**Published:** 2026-08-11

**Authors:** Nozomi Tatsuta, Yu Taniguchi, Shoji F Nakayama, Makiko Sekiyama, Mai Takagi, Miyuki Iwai-Shimada, Yayoi Kobayashi, Tomohiko Isobe, Hongwei Jiang, Shin Yamazaki, the Japan Environment and Children’s Study Group

**Affiliations:** Health and Environmental Risk Division, National Institute for Environmental Studies, Tsukuba, Ibaraki, Japan; Health and Environmental Risk Division, National Institute for Environmental Studies, Tsukuba, Ibaraki, Japan; Health and Environmental Risk Division, National Institute for Environmental Studies, Tsukuba, Ibaraki, Japan; Health and Environmental Risk Division, National Institute for Environmental Studies, Tsukuba, Ibaraki, Japan; Health and Environmental Risk Division, National Institute for Environmental Studies, Tsukuba, Ibaraki, Japan; Health and Environmental Risk Division, National Institute for Environmental Studies, Tsukuba, Ibaraki, Japan; Health and Environmental Risk Division, National Institute for Environmental Studies, Tsukuba, Ibaraki, Japan; Health and Environmental Risk Division, National Institute for Environmental Studies, Tsukuba, Ibaraki, Japan; Health and Environmental Risk Division, National Institute for Environmental Studies, Tsukuba, Ibaraki, Japan; Health and Environmental Risk Division, National Institute for Environmental Studies, Tsukuba, Ibaraki, Japan; Health and Environmental Risk Division, National Institute for Environmental Studies, Tsukuba, Ibaraki, Japan

**Keywords:** Japan Environment and Children’s Study, nationwide birth cohort study, environmental exposures, child health, child development

Key Features

- The Japan Environment and Children’s Study (JECS) is a nationwide birth cohort study that enrolled 103 099 pregnant women and 104 102 fetuses between 2011 and 2014 to investigate the effects of environmental exposures on child health and development.
- Extensive biospecimens have been collected longitudinally from pregnancy onwards and analyzed for a wide range of environmental chemicals, enabling assessment of prenatal and postnatal exposures.
- Child development and health outcomes, including birth outcomes, allergic diseases, and neurodevelopment, have been assessed using questionnaires administered twice annually, with a response rate of 78.2% at age 6, indicating good retention.
- Detailed information on a wide range of potential determinants of child development, including lifestyle and socioeconomic factors, has been systematically collected.
- Data are available to researchers upon application and approval by the JECS Data Use Committee.

## Why was the cohort set up?

Over the past 40–50 years, children’s health patterns have changed, including a decline in the secondary sex ratio [1], and increases in allergic diseases [2] and neurodevelopmental disorders such as autism spectrum disorder (ASD) [3, 4]. Although these trends may partly reflect increased maternal age, advances in diagnostic technologies, and genetic factors, they are unlikely to be explained fully by these factors alone, suggesting a potential role of environmental change.

In response, several large-scale birth cohort studies were established worldwide to examine the effects of environmental exposures on child health, including the Danish National Birth Cohort (DNBC) [5], the Norwegian Mother and Child Cohort Study (MoBa) [6], and the US National Children’s Study (NCS) [7].

In Japan, the Ministry of the Environment funded the Japan Environment and Children’s Study (JECS) in 2010 as a nationwide epidemiological study to investigate how chemicals and other environmental factors affect children’s health and development. JECS focuses on five outcome domains: pregnancy and reproductive outcomes, congenital anomalies, neurodevelopmental and psychiatric disorders, allergies and immune-related diseases, and metabolic and endocrine disorders.

JECS is conducted through a nationwide network consisting of Programme Office (PO), which provides overall coordination and data management; a Medical Support Centre (MSC), which provides medical and scientific support; and 15 Regional Centres (RCs) based at university hospitals across Japan, which are responsible for participant recruitment, data collection, and follow-up [8]. The Ministry of the Environment, Japan, serves as the governmental body responsible for planning and funding the study. Although originally designed to follow participants until age 13, the study has been extended into adolescence. Efforts are ongoing to support longer-term follow-up across the life course and into the next generation.

## Who is in the cohort?

Pregnant women in their first trimester were enrolled in JECS between January 2011 and March 2014. The Study Areas were predefined administrative regions consisting of municipalities covered by the 15 Regional Centres across Japan. Eligibility criteria were (i) residence in the Study Area with an expectation to remain in Japan for several years; (ii) an expected delivery date between August 2011 and mid-2014; and (iii) ability to complete self-administered questionnaires in Japanese. Women living outside the Study Areas were excluded, even if they attended healthcare facilities within the areas, whereas those who moved out after enrollment were followed up. Approximately 45% of births in the Study Areas were covered by JECS. Previous reports have shown that the characteristics of JECS participants closely mirrored those of the 2013 Japanese Vital Statistics [9]. Exclusion criteria included lack of consent, inability to complete questionnaires, and cases where cord blood or birth information could not be obtained.

Proxy consent for children was obtained from mothers during pregnancy, and written informed consent was obtained from pregnant women and from partners/fathers who agreed to participate. For underage parents, consent was obtained from guardians and renewed at adulthood. Participants could withdraw at any time without disadvantage. The JECS protocol was reviewed and approved by the Ministry of the Environment’s Institutional Review Board on Epidemiological Studies and the Ethics Committees of all participating institutions (Ethical Number: 100910001).

JECS consists of three tiers: the Main Study, including all parent–child pairs; the Sub-Cohort Study, a randomly selected subset of approximately 5000 pairs from the Main Study; and the Adjunct Study, investigator-initiated studies conducted at participating RCs with extramural funding. The Main Study includes questionnaire surveys, medical record-based disease ascertainment, in-person examinations, biological sample collection, and genetic analyses. The Sub-Cohort Study includes more intensive environmental measurements, medical examinations, and developmental assessments. Further details on the Sub-Cohort Study are provided by Sekiyama *et al.* [10]. Adjunct Studies are additional research projects conducted at participating RCs with approval from the Ministry of the Environment, Japan, and supported by competitive external funding. As of 30 June 2023, 197 adjunct studies had been approved.

Supplementary Figure S1 shows the geographical distribution of JECS births across Japan. In total, 103 099 pregnant women, 104 102 fetuses, and 51 909 partners/fathers were enrolled in the cohort, with partner/father participation being voluntary [2]. Participant characteristics closely matched national vital statistics, supporting the cohort’s representativeness. Currently, data through age 6 are available for analysis. After excluding withdrawals (as of 4 June 2023), the current dataset includes 103 040 pregnancies, including 988 multiple pregnancies and repeated registrations from the same mothers (5596 second and 52 third pregnancies). The dataset also contains 104 043 fetuses, including 1247 miscarriages, 389 stillbirths, and 2285 cases with missing birth information. In addition, data are available for 51 892 partners/fathers. After excluding 497 partners/fathers with missing birth information, 51 395 partners/fathers (49 170 unique partners/fathers) remained available for analysis, corresponding to 49.4% of the enrolled children. Previous analyses have shown that the characteristics of children with participating fathers did not substantially differ from those of the overall JECS population, despite paternal participation being voluntary [9].

For the present analysis, the baseline population comprised 92 767 eligible mothers. Questionnaires at the 6-year follow-up were mailed to 88 111 mothers (corresponding to 95 312 children), and responses were obtained from 68 867 mothers (corresponding to 74 577 children). The response rate was 78.2% (68 867/88 111 mothers). Table 1 presents the baseline characteristics of the overall population, as well as respondents and nonrespondents, with standardized mean differences (SMDs) used to assess differences between the groups. Response rates were similar between singleton and multiple births. Likewise, response rates were similar between families who registered only one child and those who registered siblings in JECS (Supplementary Table S1). For comparability, we focused on singletons from families registered only once in JECS and compared characteristics of respondents and nonrespondents using SMDs. Most differences were small (Table 1). Maternal age at parturition, prepregnancy body mass index, partner education, employment, marital status, household income, parental smoking during pregnancy, emotional response to pregnancy confirmation, folic acid use, late-night eating habits during pregnancy, gravidity, and infertility treatment history all had SMDs below 0.40. Maternal education and breakfast habits during pregnancy showed moderate differences, with lower response rates among participants with lower education levels and those who skipped breakfast during pregnancy. Baseline child characteristics were also similar between groups. These findings are consistent with those reported in other birth cohorts [11, 12].

**Table 1:** Baseline characteristics of the study population and comparison between respondents and nonrespondents to the 6-year questionnaire.^a^

	Baseline (*n* = 92 767)		Respondents (*n* = 68 867)		Nonrespondents (*n* = 19 244)		SMD
	*n*	%	*n*	%	*n*	%	
Maternal age at parturition (years)	31.2 ± 5.1		31.5 ± 4.9		30.1 ± 5.3		0.28
<24	9322	10.0	5440	7.9	3083	16.0	0.25
25–29	25 469	27.5	18 507	26.9	5682	29.5	
30–34	32 700	35.2	25 034	36.4	6183	32.1	
35–40	20 976	22.6	16 453	23.9	3635	18.9	
≥40	4297	4.6	3433	5.0	661	3.4	
Missing	3	0.003	−		−		
Prepregnancy body mass index (kg/m^2^)	21.2 ± 3.3		21.1 ± 3.2		21.6 ± 3.7		0.15
<18.5	15 007	16.2	11 277	16.4	2959	15.4	0.14
18.5–24.9	67 743	73.0	50 920	73.9	13 494	70.1	
≥25.0	9893	10.7	6623	9.6	2755	14.3	
Missing	124	0.1	47	0.1	36	0.2	
Maternal education (years)							
≤9	4352	4.7	2325	3.4	1642	8.5	0.40
10–12	28 420	30.6	19 990	29.0	6911	35.9	
13–16	56 438	60.8	44 730	65.0	9629	50.0	
≥17	1331	1.4	1115	1.6	145	0.8	
Missing	2226	2.4	707	1.0	917	4.8	
Partner education (years)							
≤9	6500	7.0	4072	5.9	2022	10.5	0.32
10–12	33 041	35.6	23 816	34.6	7632	39.7	
13–16	46 337	49.9	36 538	53.1	7989	41.5	
≥17	4072	4.4	3382	4.9	487	2.5	
Missing	2817	3.0	1059	1.5	1114	5.8	
Employment status							
Full-time	30 102	32.4	23 520	34.2	5562	28.9	0.17
Self-employed/family business	2885	3.1	2088	3.0	639	3.3	
Temporary/contract employee	1692	1.8	1281	1.9	352	1.8	
Homemaker/on leave	31 260	33.7	23 155	33.6	6325	32.9	
Part-time/temporary worker	18 925	20.4	13 801	20.0	4258	22.1	
Unemployed	1286	1.4	840	1.2	363	1.9	
Other	2261	2.4	1706	2.5	442	2.3	
Missing	4356	4.7	2476	3.6	1303	6.8	
Marital status							
Married (including common-law)	86 910	93.7	65 652	95.3	17 273	89.8	0.29
Never married, divorced, or widowed	4160	4.5	2635	3.8	1246	6.5	
Missing	1697	1.8	580	0.8	725	3.8	
Annual household income (million JPY)							
<4	33 877	36.5	24 400	35.4	7896	41.0	0.28
4–7.9	41 384	44.6	32 381	47.0	7404	38.5	
≥8	9224	9.9	7390	10.7	1447	7.5	
Missing	8282	8.9	4696	6.8	2497	13.0	
Maternal smoking habits during pregnancy							
Never smoker	52 916	57.0	41 973	60.9	8796	45.7	0.39
Ex-smoker (quit before pregnancy)	21 191	22.8	15 911	23.1	4276	22.2	
Quit smoking during pregnancy	12 317	13.3	7886	11.5	3696	19.2	
Current smoker	4369	4.7	2314	3.4	1703	8.8	
Missing	1974	2.1	783	1.1	773	4.0	
Partner smoking habits during pregnancy							
Never smoker	24 449	26.4	19 671	28.6	3768	19.6	0.38
Ex-smoker (quit before pregnancy)	20 787	22.4	16 558	24.0	3382	17.6	
Quit smoking during pregnancy	2273	2.5	1656	2.4	494	2.6	
Current smoker	41 941	45.2	29 397	42.7	10 401	54.0	
Missing	3317	3.6	1585	2.3	1199	6.2	
Emotional response to pregnancy confirmation							
Very happy	59 746	64.4	46 125	67.0	10 938	56.8	0.30
Surprised but happy	23 120	24.9	16 420	23.8	5564	28.9	
Surprised and confused	6281	6.8	4297	6.2	1636	8.5	
Felt troubled	517	0.6	318	0.5	166	0.9	
Felt nothing in particular	425	0.5	324	0.5	83	0.4	
Other	957	1.0	764	1.1	149	0.8	
Missing	1721	1.9	619	0.9	708	3.7	
Folic acid supplement use during pregnancy							
No usage	51 806	55.8	37 621	54.6	11 724	60.9	0.24
1–3 times a month	6803	7.3	5144	7.5	1362	7.1	
1–6 times a week	12 711	13.7	9852	14.3	2363	12.3	
Once or more a day	19 382	20.9	15 608	22.7	2942	15.3	
Missing	2065	2.2	642	0.9	853	4.4.4	
Breakfast habits during pregnancy							
Eaten every day	58 366	62.9	46 112	67.0	9838	51.1	0.40
More than once a week	17 867	19.3	12 755	18.5	4283	22.3	
Less than once a week	12 477	13.4	8091	11.7	3670	19.1	
Never eaten	1938	2.1	1252	1.8	564	2.9	
Missing	2119	2.3	657	1.0	889	4.6	
Late-night snacking habits during pregnancy							
Eaten every day	3836	4.1	2783	4.0	852	4.4	0.23
More than once a week	16 199	17.5	11 732	17.0	3660	19.0	
Less than once a week	28 062	30.2	20 729	30.1	6112	31.8	
Never eaten	42 532	45.8	32 962	47.9	7722	40.1	
Missing	2138	2.3	661	1.0	898	4.7	
Gravidity							
Primigravida	29 918	32.3	23 489	34.1	5082	26.4	0.23
Multigravida	60 933	65.7	44 595	64.8	13 434	69.8	
Missing	1916	2.1	783	1.1	728	3.8	
Infertility treatment							
Yes	8158	8.8	6772	9.8	1048	5.4	0.28
No	82 892	89.4	61 481	89.3	17 497	90.9	
Missing	1717	1.9	614	0.9	699	3.6	
Gestational age (days)	274.7 ± 11.1		274.9 ± 10.8		274.5 ± 11.1		0.04
Child sex							
Male	47 588	51.3	35 339	51.3	9817	51.0	0.01
Female	45 168	48.7	33 528	48.7	9427	49.0	
Unknown	11	0.01	−		−		
One-minute Apgar score							
0–3	579	0.6	375	0.5	105	0.5	0.03
4–7	3435	3.7	2482	3.6	709	3.7	
8–10	86 581	93.3	64 351	93.4	18 037	93.7	
Missing	2172	2.3	1659	2.4	393	2.0	
5-min Apgar score							
0–3	167	0.2	98	0.1	32	0.2	0.02
4–7	882	1.0	585	0.8	172	0.9	
8–10	87 122	93.9	64 771	94.1	18 093	94.0	
Missing	4596	5.0	3413	5.0	947	4.9	
Delivery type							0.04
Vaginal delivery	75 017	80.9	55 985	81.3	15 339	79.7	
Cesarean section	17 503	18.9	12 712	18.5	3849	20.0	
Missing	247	0.3	170	0.2	56	0.3	
Birth weight (g)	3021 ± 420.3		3024 ± 413.0		3023 ± 422.1		0.002

a The 6-year questionnaire was mailed to 88 111 mothers, corresponding to 95 312 children.

Although overall differences were small, potential attrition bias should be considered. These findings highlight the need to maintain participant retention and to apply appropriate methods, such as imputation or weighting, in analyses.

## How often have they been followed up?

Questionnaire surveys were administered during pregnancy, at 1 month postpartum, and every 6 months thereafter. During the preschool period, surveys were conducted on each child’s birthday and again 6 months later. After school entry, questionnaires were administered on birthdays and once again within the same academic year. From age 10 onwards, children completed self-administered questionnaires in addition to parental questionnaires. Survey schedules are summarized in Table 2. At age 6, the questionnaire response rate was 78.2%, which was higher than those reported in the DNBC (63.0%) and MoBA (54.2%) [12].

**Table 2:** Survey schedule and response rates for the questionnaires.

	Survey period^a^	Main Study						Sub-Cohort Study		
		Questionnaire response rate	Maternal questionnaire	Child self-administered questionnaire	In-person examination	Deciduous teeth	Medical questionnaires	Environmental measurement	Medical examination	Developmental test^b^
First trimester	2011–14	98.1	✓							
Second/third trimester	2011–14	98.8	✓							
1 month	2011–14	97.7	✓							
6 months	2011–14	94.2	✓							
12 months	2012–15	91.4	✓							
18 months	2012–15	89.3	✓					✓		
24 months	2013–16	87.4	✓				✓		✓	✓
30 months	2013–16	85.7	✓					✓		
3 years	2014–17	84.3	✓							
3.5 years	2014–17	81.8	✓							
4 years	2015–18	80.6	✓						✓	✓
4.5 years	2015–18	78.9	✓							
5 years	2016–19	76.9	✓							
5.5 years	2016–19	78.1	✓							
6 years	2017–20	78.3	✓						✓	
7 years	2018–21	78.9	✓				✓			
1st grade	2018–21	80.3	✓							
8 years	2019–22	78.4	✓						✓	✓
2nd grade	2019–22	77.6	✓		✓					
9 years	2020–23	77.0	✓							
3rd grade	2020–23	76.6	✓							
10 years	2021–24	73.0	✓	✓					✓	✓
4th grade	2021–24	74.4	✓			✓				
11 years	2022–25	70.8	✓	✓						
5th grade	2022–25	71.0	✓							
12 years	2023–26	64.6	✓	✓			✓		✓	✓
6th grade	2023–26	64.9	✓		✓					

a As of 1 November 2025.

b Kyoto Scale of Psychological Development 2001 at ages 2 and 4 years; Computer Assisted Tests (Finger Tapping Test, Continuous Performance Test, and Estimation Line) at ages 8 and 12 years; and the Wechsler Intelligence Scale for Children-Fourth Edition (WISC-IV) at age 10 years.

In-person examinations were conducted in the second and sixth grades, collecting data on physical growth and development, including anthropometric measurements, developmental tests, skin examinations, and urine tests. Blood sampling was additionally performed in the sixth grade.

In the Sub-Cohort Study, medical examinations were conducted at ages 2, 4, 6, 8, and 10, with additional follow-up assessments currently ongoing at age 12. Developmental tests administered at each follow-up are shown in Table 2.

## What has been measured?

### Biological and environmental samples

JECS collected biological and environmental samples from mothers, partners/fathers, and children (Table 3). These samples were used for environmental exposure assessment, including measurements of metals and environmental pollutants (Table 4). The large-scale, comprehensive measurement programme enables precise evaluation of associations between chemical exposures and child health. To evaluate potential selection bias related to chemical exposure, Supplementary Table S2 compares concentrations of heavy metals, pesticides, and cotinine during pregnancy between respondents and nonrespondents to the age 6 questionnaire. For pesticide concentrations below the method detection limit (MDL), half the MDL was assigned; values between the MDL and the minimum reporting level were replaced with their average. Differences were evaluated using SMDs. Nonrespondents tended to have higher levels of lead, cadmium, manganese, and cotinine, whereas respondents had higher pesticide levels. However, most differences were negligible (SMD < 0.1), except for cotinine (SMD = 0.403), consistent with the lower response rates among women who smoked during pregnancy.

**Table 3:** Details of the collected biological and environmental samples.^a^

Biomarker		Sampling period	Sample volume	Sampling period	Study type	Number of collected samples
Blood	Mother	1st trimester	32 ml	2011–14	Main Study	89 365
		2nd–3rd trimester	33 ml	2011–14	Main Study	95 235
		At birth	18 ml	2011–14	Main Study	93 633
	Father		32 ml	2011–14	Main Study	49 881
	Cord blood		35 ml	2011–14	Main Study	86 793
	Child	At birth	Dried blood spots	2011–14	Main Study	93 787
		2-year-old	4 ml	2015–16	Sub-Cohort Study	4728
		4-year-old	4 ml	2017–18	Sub-Cohort Study	4231
		6-year-old	10 ml	2019–20	Sub-Cohort Study	3368
		8-year-old	10 ml	2021–22	Sub-Cohort Study	3273
		10-year-old	10 ml	2023–24	Sub-Cohort Study	3158
		12-year-old	10 ml	2025–26	Sub-Cohort Study	Ongoing
		6th grade	10 ml	2023–26	Main Study	Ongoing
Urine	Mother	1st trimester	35 ml	2011–14	Main Study	89 152
		2nd–3rd trimester	25 ml	2011–14	Main Study	95 497
	Child	4-year-old	20 ml	2017–18	Sub-Cohort Study	4283
		6-year-old	20 ml	2019–20	Sub-Cohort Study	3606
		8-year-old	20 ml	2021–22	Sub-Cohort Study	3552
		10-year-old	10 ml	2023–24	Sub-Cohort Study	3551
		12-year-old	10 ml	2025–26	Sub-Cohort Study	Ongoing
		2nd grade	20 ml	2019–22	Main Study	36 921
		6th grade	10 ml	2023–26	Main Study	Ongoing
Breast milk	Mother	1 month	20 ml	2011–14	Main Study	88 404
Hair	Mother	At birth	1 mg	2011–14	Main Study	95 127
	Infant			2011–14	Main Study	95 301
Deciduous teeth	Child	4th grade	2 teeth	2020–	Main Study	29 866 (ongoing)
Indoor/outdoor air		1.5-year-old		2014–16	Sub-Cohort Study	5009
		3-year-old		2016-2018	Sub-Cohort Study	4712
House dust		1.5-year-old	dust collected from the mattress/futon	2014–16	Sub-Cohort Study	5009
Vacuum dust		3-year-old	dust collected from vacuum cleaner bag	2014–16	Sub-Cohort Study	4920

a Preliminary value as of 10 November 2025.

**Table 4:** Details of the measured chemical exposures.

Biomarker		Sampling period	Chemical substances	Measurement year	Number of samples analyzed	Notes
Blood	Mother	Pregnancy	Metals (Pb, Cd, Hg, Mn, Se)	2014–17	95 811	
			PFAS	2017	25 000	
			Aryl hydrocarbon receptor activity	2020	4 956	
			Persistent organic pollutants (PCBs, DDTs, PBDEs)	2020	13 000	
			Acrylamide	2023	5 000	
			Synthetic fragrances (nitro musks, polycyclic musks)	2025	5 000	Ongoing
	Father	Pregnancy, Sub-Cohort Study	Metals (Pb, Cd, Hg, Mn, Se)	2022–23	2 500	
	Cord-blood	At delivery	Metals (Pb, Cd, Hg, Mn, Se)	2018	3 897	
		At delivery	Metals (MeHg, IHg)	2018	3 897	
		At delivery	PFAS	2020	5 001	
	Child	2-year-old: Sub-Cohort Study	Metals (Pb, Cd, Hg, Mn, Se, Cu, Zn, Mg, Ca)	2025	8 000	Ongoing
		4-year-old: Sub-Cohort Study	PFAS	2021	5 010	
		8-year-old: Sub-Cohort Study	PFAS	2024	5 075	
Urine	Mother	Pregnancy	Cotinine, 8-OHdG	2014–17	96 490	
			Phenols	2018	10 000	
			Organophosphate pesticide metabolites	2018	5727	
			Phthalate ester metabolites	2018–19	19 999	
			Neonicotinoid pesticides	2019	20 000	
			Arsenic species	2020	5 039	
			Pyrethroid pesticide metabolites	2021	10 013	
			Pesticides and repellents	2022	5 000	
			Organophosphate flame retardants	2022–23	10 000	
	Child	8-year-old: Sub-Cohort Study	Cotinine	2023	10 000	
		2nd-grade school-age examination				
Deciduous teeth	Child	4th grade	Metals	2020–	Ongoing	
Indoor/outdoor air		1.5-year-old: Sub-Cohort Study	Volatile organic compounds (benzene, toluene, ethylbenzene, xylene, styrene, p-dichlorobenzene)	2014–16	5 006/4 990	
			Aldehydes (formaldehyde, acetaldehyde)	2014–16	5 005/4 993	
			Nitrogen dioxide, sulfur dioxide, ozone	2014–2016	5 006/4 992	
			PM_2.5_	2014–16	5 006/4 993	
		3-year-old: Sub-Cohort Study	Volatile organic compounds (benzene, toluene, ethylbenzene, xylene, styrene, *p*-dichlorobenzene)	2016–18	4 711/4 711	
			Aldehydes (formaldehyde, acetaldehyde)	2016–18	4 711/4 711	
			Nitrogen dioxide, sulfur dioxide, ozone	2016–18	4 711/4 711	
			PM_2.5_	2016–18	4711/4711	
House dust		1.5-year-old: Sub-Cohort Study	Mite allergen/endotoxin	2014–16	5009	

8-OHdG, 8-hydroxy-2ʹ-deoxyguanosine; Cd, cadmium; DDT, dichlorodiphenyltrichloroethane; Hg, mercury; IHg, inorganic mercury; MeHg, methylmercury; Mn, manganese; PBDE, polybrominated diphenyl ethers; PCB, polychlorinated biphenyls; PFAS, per- and polyfluoroalkyl substances; PM, particulate matter; Pb, lead; Se, selenium.

These biospecimens were also used for clinical biochemical analyses, including thyroid function (TSH), vitamin D status [25(OH)D], growth-related hormones, and renal markers such as creatinine, as summarized in Table 5. Blood, urine, and hair were obtained during pregnancy; cord blood, dried blood spots, and hair at birth; and breast milk at 1 month postpartum. Children’s urine was collected in grades 2 and 6, and blood in grade 6. In the Sub-Cohort Study, blood samples were collected every 2 years from age 2, whereas urine samples were collected every 2 years starting at age 4. Blood, urine, and breast milk are stored at −80°C, whereas other biospecimens are stored at controlled room temperature. These biospecimens are stored in secure biorepositories and are available for comprehensive chemical and biomarker analyses. Detailed procedures for biospecimen collection, storage, and quality control have been described previously in the JECS protocol paper [8]. Chemical analyses were conducted in laboratories adhering to ISO/IEC 17025 standards, whereas biochemical analyses were performed in laboratories accredited by the College of American Pathologists and compliant with ISO 15189 standards.

**Table 5:** Details of the measured biochemical tests.

	2-year-old: Sub-Cohort Study	4-year-old: Sub-Cohort Study	6-year-old: Sub-Cohort Study
Plasma	4872	4437	3673
Specific IgE	DCP microarray	DCP microarray	DCP microarray
nonspecific IgE	FEIA method	FEIA method	FEIA method
TSH	CLIA method	CLIA method	ECLIA method
FT4	CLIA method	CLIA method	ECLIA method
25(OH)D_2_	LC-MS/MS method	LC-MS/MS method	LC-MS/MS method
25(OH)D_3_	LC-MS/MS method	LC-MS/MS method	LC-MS/MS method
IGF-1	–	–	ECLIA method
Creatinine	–	–	Enzymatic method
Urine		4426	3669
Gravity	–	Refractometric method	Refractometric method
Creatinine	–	Enzymatic method	Enzymatic method

25(OH)D, 25-hydroxyvitamin D; CLIA, chemiluminescent immunoassay; DCP, densely carboxylated protein; ECLIA, electrochemiluminescence immunoassay; FEIA, fluorescent enzyme immunoassay; FT4, free thyroxine 4; IGF-1, insulin-like growth factor 1; LC-MS/MS, liquid chromatography–tandem mass spectrometry; TSH, thyroid-stimulating hormone.

### Questionnaire

Self-administered questionnaires were conducted during the first and second/third trimesters to collect information on demographics, health, lifestyle, occupation, environmental exposure, and socioeconomic status. Medical record transcriptions from pregnancy and postpartum provided data on medical history, pregnancy complications, and infant outcomes. Details of the questionnaires are described by Michikawa *et al.* [13]. Age-appropriate questionnaires were also used to assess neurodevelopmental and psychiatric disorders, allergies, and metabolic and endocrine disorders. In addition, information on potential confounders, including home environment, lifestyle, occupation, mental health, and diet, was collected. Participating partners/fathers also completed a questionnaire during the mother’s pregnancy. From age 10, children completed questionnaires on school environment and subjective well-being. Table 6 summarizes the main questionnaire variables/categories and developmental assessment tools across the five outcome domains assessed in JECS.

**Table 6:** Main questionnaire variables/categories and developmental assessment tools across the five outcome domains.

Outcome domain	Main variables/categories	Assessment tools/data source
Pregnancy and reproductive outcomes	Miscarriage, stillbirth, gestational age, birth weight, preterm birth, sex, delivery type, parity, pregnancy complications	Medical records/questionnaires
Congenital anomalies	Hypospadias, cryptorchidism, cleft lip/palate, gastrointestinal atresia, ventricular septal defect	Questionnaire/medical records
Neurodevelopmental and psychiatric disorders	Developmental milestones (motor, language, social), ASD, ADHD traits/diagnosis, behavioral problems	ASQ, ESSENCE-Q, SRS-P, SDQ (questionnaires)/medical records
Allergies and immune-related diseases	Asthma, atopic dermatitis, allergic rhinitis, food allergy	ISAAC-based questionnaire
Metabolic and endocrine disorders	Childhood obesity, growth trajectories, thyroid-related conditions, metabolic abnormalities	Questionnaire/medical records
Other outcomes	Cancer, infectious diseases, hospitalizations, chronic conditions not classified above	Questionnaire/medical records

ASQ, Ages & Stages Questionnaires; SDQ, Strengths and Difficulties Questionnaire; SRS-P, Social Responsiveness Scale Preschool version; ISAAC, International Study of Asthma and Allergies in Childhood.

### Developmental tests in the second- and sixth-grade in-person examinations

In in-person examinations, second- and sixth-grade children completed two subtests: the Conners’ CPT 3rd edition [14], and the FTT. The CPT assesses attention and is used to screen for ADHD [14], while the FTT measures finger dexterity. Both are widely used in epidemiological studies and allow objective assessment at scale [15, 16]. Previous studies have linked mercury and lead exposure to CPT and FTT performance [16, 17]. JECS uses these tests to examine the effects of chemical exposure in Japanese children.

## What has it found?

Nearly 600 peer-reviewed papers have been published using JECS data over the past decade. This output reflects frequent questionnaire follow-up, reporting of both positive and null findings, collaboration across many universities and institutions, and active involvement of early-career researchers. To date, no chemicals requiring immediate regulatory action have been identified.

### Effects of chemicals

Per- and poly-fluoroalkyl substances (PFAS) have attracted global attention, although epidemiological evidence remains limited. In JECS, 28 PFAS were measured in 25 000 pregnant women. No associations were observed with late miscarriage, and inverse associations were found with Kawasaki disease and asthma at age 4 [18–20]. On the other hand, perfluorononanoic acid and perfluorooctanesulfonic acid were associated with an increased risk of chromosomal abnormalities, although potential collider bias cannot be excluded [21]. Ongoing analyses of children’s blood samples will help clarify the effects of both prenatal and current exposure.

JECS has also evaluated heavy metals such as lead. Although maternal blood lead levels have declined in Japan, even low levels were associated with an increased proportion of male births [22] and decreased birth weight [23]. No associations were observed with premature birth, congenital malformations, Kawasaki disease, or developmental delay [24–29]. Child blood measurements are ongoing.

### Effects of other environmental factors

JECS also examines broader environmental exposures. Findings that have attracted particular attention include associations of pet ownership and child development [30–33], and of screen time and ASD or sleep outcomes [34, 35].

Smoking during pregnancy has been linked to adverse outcomes, including reduced infant body size [36–38], increased obesity risk at age 3 [39], asthma [40], and congenital anomalies such as trisomy [41]. The Healthy Parents and Children 21 initiative by the Ministry of Health, Labour and Welfare aims to eliminate smoking during pregnancy, and the smoking rate has declined. However, the goal of achieving zero smoking during pregnancy has not yet been achieved. JECS findings may help inform continued efforts toward that goal.

## What are the main strengths and weaknesses?

The main strengths of JECS are its representativeness, relatively high retention, extensive biospecimen collection, and rich covariate data for bias adjustment. In 2013, approximately 45% of children in the Study Areas were registered, and participant characteristics closely mirrored national statistics [2]. Health data were obtained from questionnaires and medical record transcriptions, with major outcomes confirmed by physicians. These extensive exposome data support the evaluation of multiple exposures and more robust control of bias.

Nevertheless, the study has several limitations. Partner data are limited, with only about half of the partners enrolled, despite known paternal influences. Recruitment began around 12 weeks of gestation, so data on early miscarriage and preconception exposure are limited. In addition, response rates declined over time, and some differences were observed between participants who remained and those who dropped out.

A key challenge is maintaining long-term retention. Follow-up has been extended beyond age 13, requiring renewed consent and a shift to online questionnaires, both of which may reduce response rates. Sustaining engagement, particularly during adolescence, is essential for the cohort’s continuation.

## Can I get hold of the data? Where can I find out more?

Further information on JECS is available at https://www.env.go.jp/chemi/ceh/en/index.html. As of April 2026, 597 peer-reviewed papers had been published, mainly using Main Study data. Results from the Sub-Cohort Study, including analysis of environmental exposure, developmental testing, and medical examinations, are expected to be reported soon. All inquiries regarding data access should be sent to jecs-en@nies.go.jp. Dr Shoji F. Nakayama (JECS Programme Office, National Institute for Environmental Studies) is responsible for handling enquiries related to the JECS data resource.

## Ethics approval

This study was conducted in accordance with the Declaration of Helsinki. The JECS protocol was reviewed and approved by the Japan Ministry of the Environment’s Institutional Review Board on Epidemiological Studies and Ethics Committees of all participating institutions (Ethical Number: 100910001). Written informed consent was obtained from all participating mothers and from fathers who agreed to participate. Age-appropriate explanations of the examinations, including blood collection procedures, were provided to the children, and the examinations were conducted only when they agreed to participate. In accordance with the study protocol, informed consent will be obtained from participants themselves after they reach adulthood.

## Supplementary Material

dyag146_Supplementary_Data

## Data Availability

Data are unsuitable for public deposition due to ethical restrictions and legal framework of Japan. It is prohibited by the Act on the Protection of Personal Information (Act No. 57 of 30 May 2003, amendment on 9 September 2015) to public deposition the data containing personal information. Ethical Guidelines for Medical and Health Research Involving Human Subjects enforced by the Japan Ministry of Education, Culture, Sports, Science and Technology and the Ministry of Health, Labour and Welfare also restrict the open sharing of the epidemiologic data. All inquiries about access to data should be sent to: jecs-en@nies.go.jp. The person responsible for handling enquiries sent to this e-mail address is Dr Shoji F. Nakayama, JECS Programme Office, National Institute for Environmental Studies.

## References

[1] Davis DL , Webster P, Stainthorpe H et al Declines in sex ratio at birth and fetal deaths in Japan, and in U.S. Whites but not African Americans. Environ Health Perspect 2007;115:941–6. 10.1289/ehp.9540. 17589604 PMC1892130

[2] Asher MI , Montefort S, Björkstén B et al; ISAAC Phase Three Study Group. Worldwide time trends in the prevalence of symptoms of asthma, allergic rhinoconjunctivitis, and eczema in childhood: ISAAC Phases One and Three repeat multicountry cross-sectional surveys. Lancet 2006;368:733–43. 10.1016/S0140-6736(06)69283-0. 16935684

[3] Talantseva OI , Romanova RS, Shurdova EM et al The global prevalence of autism spectrum disorder: a three-level meta-analysis. Front Psychiatry 2023;14:1071181. 10.3389/fpsyt.2023.1071181. 36846240 PMC9947250

[4] Williams JG , Higgins JP, Brayne CE. Systematic review of prevalence studies of autism spectrum disorders. Arch Dis Child 2006;91:8–15. 10.1136/adc.2004.062083. Epub 2005 Apr 29. 15863467 PMC2083083

[5] Ernst A , Brix N, Lauridsen LLB et al Cohort profile: the puberty cohort in the Danish National Birth Cohort (DNBC). Int J Epidemiol 2020;49:373–374g. 10.1093/ije/dyz222. 31697338 PMC7266555

[6] Magnus P , Irgens LM, Haug K et al; MoBa Study Group. Cohort profile: the Norwegian Mother and Child Cohort Study (MoBa). Int J Epidemiol 2006;35:1146–50. 10.1093/ije/dyl170. 16926217

[7] Branum AM , Collman GW, Correa A et al; National Children’s Study Interagency Coordinating Committee, U.S. Environmental Protection Agency. The National Children’s Study of environmental effects on child health and development. Environ Health Perspect 2003;111:642–6. 10.1289/ehp.111-1241458. 12676629 PMC1241458

[8] Kawamoto T , Nitta H, Murata K et al Rationale and study design of the Japan environment and children’s study (JECS). BMC Public Health 2014;14:25. 10.1186/1471-2458-14-25. 24410977 PMC3893509

[9] Michikawa T , Nitta H, Nakayama SF et al; Japan Environment and Children’s Study Group. Baseline profile of participants in the Japan Environment and Children’s Study (JECS). J Epidemiol 2018;28:99–104. 10.2188/jea.JE20170018. 29093304 PMC5792233

[10] Sekiyama M , Yamazaki S, Michikawa T et al; Japan Environment and Children’s Study Group. Study design and participants’ profile in the Sub-Cohort Study in the Japan Environment and Children’s Study (JECS). J Epidemiol 2022;32:228–36. 10.2188/jea.JE20200448. 33390465 PMC8979916

[11] Charles MA , Thierry X, Lanoe JL et al Cohort Profile: The French national cohort of children (ELFE): birth to 5 years. Int J Epidemiol 2020;49:368–369j. 10.1093/ije/dyz227. 31747017 PMC7266552

[12] Vejrup K , Magnus P, Magnus M. Lost to follow-up in the Norwegian mother, father and child cohort study. Paediatr Perinat Epidemiol 2022;36:300–9. 10.1111/ppe.12821. 34797579

[13] Michikawa T , Nitta H, Nakayama SF et al; Japan Environment and Children’s Study Group. The Japan Environment and Children’s Study (JECS): a preliminary report on selected characteristics of approximately 10000 pregnant women recruited during the first year of the study. J Epidemiol 2015;25:452–8. 10.2188/jea.JE20140186. 25912098 PMC4444500

[14] Conners CK , Conners Continuous Performance Test. 3rd edition manual. Toronto: MHS Inc., 2014.

[15] Walkowiak J , Altmann L, Krämer U et al Cognitive and sensorimotor functions in 6-year-old children in relation to lead and mercury levels: adjustment for intelligence and contrast sensitivity in computerized testing. Neurotoxicol Teratol 1998;20:511–21. 10.1016/s0892-0362(98)00010-5. 9761589

[16] Grandjean P , Weihe P, White RF et al Cognitive deficit in 7-year-old children with prenatal exposure to methylmercury. Neurotoxicol Teratol 1997;19:417–28. 10.1016/s0892-0362(97)00097-4. 9392777

[17] Ruebner RL , Hooper SR, Parrish C et al Environmental lead exposure is associated with neurocognitive dysfunction in children with chronic kidney disease. Pediatr Nephrol 2019;34:2371–9. 10.1007/s00467-019-04306-7. 31327061 PMC6800774

[18] Iwata H , Kobayashi S, Itoh M et al The association between prenatal per-and polyfluoroalkyl substance levels and Kawasaki disease among children of up to 4 years of age: a prospective birth cohort of the Japan Environment and Children’s study. Environ Int 2024;183:108321. 10.1016/j.envint.2023.108321. 38061246

[19] Atagi T , Hasegawa K, Motoki N et al Associations between prenatal exposure to per- and polyfluoroalkyl substances and wheezing and asthma symptoms in 4-year-old children: the Japan Environment and Children’s Study. Environ Res 2024;240(Pt 1):117499. 10.1016/j.envres.2023.117499. 37914018

[20] Tatsuta N , Iwai-Shimada M, Isobe T et al Association between exposure to perfluoroalkyl compounds during early pregnancy and risk of late miscarriage: the Japan Environment and Children’s Study. Int J Hyg Environ Health 2025;270:114673. 10.1016/j.ijheh.2025.114673. 40997370

[21] Hasegawa K , Motoki N, Inaba Y et al; Japan Environment and Children’s Study Group. Maternal exposure to per- and polyfluoroalkyl substances and offspring chromosomal abnormalities: the Japan Environment and Children’s Study. Environ Health Perspect 2024;132:97004. 10.1289/EHP13617. 39258902 PMC11389478

[22] Tatsuta N , Asato K, Anai A et al; Japan Environment and Children’s Study Group. Timing of maternal smoking cessation and newborn weight, height, and head circumference. Obstet Gynecol 2023;141:119–25. 10.1097/AOG.0000000000004991. 36701612

[23] Goto Y , Mandai M, Nakayama T et al Association of prenatal maternal blood lead levels with birth outcomes in the Japan Environment and Children’s Study (JECS): a nationwide birth cohort study. Int J Epidemiol 2021;50:156–64. 10.1093/ije/dyaa162. 33141187

[24] Tsuji M , Shibata E, Morokuma S et al The association between whole blood concentrations of heavy metals in pregnant women and premature births: the Japan Environment and Children’s Study (JECS). Environ Res 2018;166:562–9. 10.1016/j.envres.2018.06.025. 29966876

[25] Miyashita C , Saijo Y, Ito Y et al Association between the concentrations of metallic elements in maternal blood during pregnancy and prevalence of abdominal congenital malformations: the Japan Environment and Children’s Study. Int J Environ Res Public Health 2021;18:10103. 10.3390/ijerph181910103. 34639405 PMC8507911

[26] Takeuchi M , Yoshida S, Kawakami C et al; Japan Environment and Children’s Study Group. Association of maternal heavy metal exposure during pregnancy with isolated cleft lip and palate in offspring: Japan Environment and Children’s Study (JECS) cohort study. PLoS One 2022;17:e0265648. 10.1371/journal.pone.0265648. 35324965 PMC8947080

[27] Ikeda A , Marsela M, Miyashita C et al; Japan Environment and Children’s Study Group. Heavy metals and trace elements in maternal blood and prevalence of congenital limb abnormalities among newborns: the Japan Environment and Children’s Study. Environ Health Prev Med 2024;29:36. 10.1265/ehpm.23-00366. 39048352 PMC11273044

[28] Yanai T , Yoshida S, Takeuchi M et al; Japan Environment and Children’s Study Group. Association between maternal heavy metal exposure and Kawasaki Disease, the Japan Environment and Children’s Study (JECS). Sci Rep 2024;14:9947. 10.1038/s41598-024-60830-z. 38689029 PMC11061304

[29] Inoue H , Sanefuji M, Sonoda Y et al; Japan Environment and Children’s Study Group. No association between prenatal lead exposure and neurodevelopment during early childhood in the Japan Environment and Children’s Study. Sci Rep 2022;12:15305. 10.1038/s41598-022-19509-6. 36097036 PMC9468004

[30] Minatoya M , Araki A, Miyashita C et al Cat and dog ownership in early life and infant development: a prospective birth cohort study of Japan Environment and Children’s Study. Int J Environ Res Public Health 2019;17:205. 10.3390/ijerph17010205. 31892205 PMC6981655

[31] Motoki N , Inaba Y, Toubou H et al; Japan Environment and Children’s Study (JECS) Group. Impact of dog and/or cat ownership on functional constipation at 3 years of age: the Japan Environment and Children’s study. BMC Pediatr 2023;23:595. 10.1186/s12887-023-04412-4. 37996790 PMC10666348

[32] Okabe H , Hashimoto K, Yamada M et al; Japan Environment and Children’s Study (JECS). Associations between fetal or infancy pet exposure and food allergies: the Japan Environment and Children’s Study. PLoS One 2023;18:e0282725. 10.1371/journal.pone.0282725. 36989214 PMC10057762

[33] Shirato K , Oba K, Matsuyama Y et al; Japan Environment and Children’s Study (JECS) Group. Association of longitudinal pet ownership with wheezing in 3-year-old children using the distributed lag model: the Japan Environment and Children’s Study. Environ Health 2024;23:53. 10.1186/s12940-024-01087-x. 38844911 PMC11155167

[34] Kushima M , Kojima R, Shinohara R et al; Japan Environment and Children’s Study Group. Association between screen time exposure in children at 1 year of age and autism spectrum disorder at 3 years of age: the Japan Environment and Children’s Study. JAMA Pediatr 2022;176:384–91. 10.1001/jamapediatrics.2021.5778. 35099540 PMC8804971

[35] Nishioka T , Hasunuma H, Okuda M et al Effects of screen viewing time on sleep duration and bedtime in children aged 1 and 3 years: Japan Environment and Children’s Study. Int J Environ Res Public Health 2022;19:3914. 10.3390/ijerph19073914. 35409594 PMC8997461

[36] Suzuki K , Shinohara R, Sato M et al Association between maternal smoking during pregnancy and birth weight: an appropriately adjusted model from the Japan Environment and Children’s Study. J Epidemiol 2016;26:371–7. 10.2188/jea.JE20150185. 26902166 PMC4919482

[37] Shiohama T , Hisada A, Yamamoto M et al; Japan Environment Children’s Study (JECS) Group. Decreased head circumference at birth associated with maternal tobacco smoke exposure during pregnancy on the Japanese prospective birth cohort study. Sci Rep 2021;11:18949. 10.1038/s41598-021-98311-2. 34556740 PMC8460647

[38] Tatsuta N , Nakai K, Nakayama SF et al Effects of maternal exposure to lead on secondary sex ratio in Japan: the Japan Environment and Children’s Study. Sci Total Environ 2022;817:152726. 10.1016/j.scitotenv.2021.152726. 34995582

[39] Horiuchi S , Shinohara R, Otawa S et al Influence of maternal active and secondhand smoking during pregnancy on childhood obesity at 3 years of age: a nested case-control study from the Japan Environment and Children’s Study (JECS). Int J Environ Res Public Health 2021;18:12506. 10.3390/ijerph182312506. 34886230 PMC8657368

[40] Miyake K , Kushima M, Shinohara R *et al.*; Japan Environment and Children’s Study Group. Maternal smoking status before and during pregnancy and bronchial asthma at 3 years of age: a prospective cohort study. Sci Rep 2023;13:3234. 10.1038/s41598-023-30304-9. 36828882 PMC9958124

[41] Tsuchida A , Hamazaki K, Kigawa M et al; and the Japan Environment and Children’s Study Group. Association between maternal smoking history and congenital anomalies in children: results from the Japan Environment and Children’s Study. Congenit Anom (Kyoto) 2021;61:159–68. 10.1111/cga.12430. 34041797 PMC8453515

