# Supplementary material for: Cohort profile: The Japan Environment and Children’s Study
Source: Int J Epidemiol. 2026 Aug 11;55(4):dyag146. doi: 10.1093/ije/dyag146 (PMC13457085; doi:10.1093/ije/dyag146)
Supplement: Supplementary file 1 [file dyag146_supplementary_data.docx]

Supplementary Figure 1. Geographic distribution of childbirth locations among pregnant women registered in the JECS

Orange dots indicate the locations where pregnant women delivered their infants, and red dots indicate the locations of the Regional Centres.

| Supplementary Table 1. Comparison of respondent status by birth type and number of enrolments | | | | | | | | | | | | |
| --- | --- | --- | --- | --- | --- | --- | --- | --- | --- | --- | --- | --- |
|  |  | Participants enrolled |  | Questionnaires mailed |  | Respondents | |  | Non-respondents | |  | SMD |
|  |  |  |  |  |  | n | % |  | n | % |  |  |
|  |  | 104,043 |  | 95,312 |  | 74,577 | 78.2 |  | 20,735 | 21.8 |  |  |
| Birth type | |  |  |  |  |  |  |  |  |  |  | 0.006 |
|  | Singleton | 102,049 |  | 93,565 |  | 73,197 | 78.2 |  | 20,368 | 21.8 |  |  |
|  | Multiple birth | 1,994 |  | 1,747 |  | 1,380 | 79.0 |  | 367 | 21.0 |  |  |
| Number of enrolments | |  |  |  |  |  |  |  |  |  |  | 0.003 |
|  | Single-enrolment mothers | 103,040 |  | 94,443 |  | 73,892 | 78.2 |  | 20,551 | 21.8 |  |  |
|  | Multiple-enrolment mothers | 1,003 |  | 869 |  | 685 | 78.8 |  | 184 | 21.2 |  |  |
| SMD, standardized mean difference | | |  |  |  |  |  |  |  |  |  |  |

| Supplementary Table 2. Differences in exposure levels to chemical substances between respondents and non-respondents in the six-year questionnaire | | | | | | | | | | | | | | | |
| --- | --- | --- | --- | --- | --- | --- | --- | --- | --- | --- | --- | --- | --- | --- | --- |
|  |  |  |  |  |  |  |  |  |  |  |  |  |  |  |  |
|  |  | Respondents | | | |  | | Non-Respondents | | | |  | | SMD | |
|  |  | n | Median (95%tile) | |  | | n | | Median (95%tile) | |  | |  | |  |
| Maternal blood (second/third trimester) | | | | | | | | | | | | | | | |
|  | Mercury | 62,395 | 3.64 | (1.51–8.78) |  | | 21,649 | | 3.53 | (1.41–8.79) |  | | 0.045 | |  |
|  | Lead | 62,395 | 5.82 | (3.44–10.6) |  | | 21,649 | | 5.92 | (3.48–10.7) |  | | 0.034 | |  |
|  | Cadmium | 62,395 | 0.66 | (0.33–1.44) |  | | 21,649 | | 0.67 | (0.33–1.55) |  | | 0.041 | |  |
|  | Manganese | 62,395 | 15.3 | (9.5–24.4) |  | | 21,649 | | 15.5 | (9.4–24.9) |  | | 0.044 | |  |
|  | Selenium | 62,395 | 168 | (141–205) |  | | 21,649 | | 169 | (141–206) |  | | 0.044 | |  |
| Maternal urine (second/third trimester) | | | | | | | | | | | | | | | |
|  | Cotinine | 62,569 | 0.13 | (0.03–176.5) |  | | 21,662 | | 0.29 | (0.03–1340) |  | | 0.403 | |  |
| Maternal urine (first trimester) | | | | | | | | | | | | | | | |
|  | Acetamiprid | 7,158 | 0.001 | (0.001–0.055) |  | | 1,659 | | 0.001 | (0.001–0.058) |  | | 0.024 | |  |
|  | Acetamipride-N-desmethyl | 7,158 | 0.313 | (0.029–3.660) |  | | 1,659 | | 0.267 | (0.029–2.850) |  | | 0.081 | |  |
|  | Clothianidin | 7,158 | 0.126 | (0.011–1.300) |  | | 1,659 | | 0.129 | (0.016–1.400) |  | | 0.018 | |  |
|  | Clothianidin-desmethyl | 7,158 | 0.073 | (0.047–0.392) |  | | 1,659 | | 0.073 | (0.047–0.404) |  | | 0.012 | |  |
|  | Dinotefuran | 7,158 | 0.198 | (0.010–7.360) |  | | 1,659 | | 0.192 | (0.010–6.640) |  | | 0.016 | |  |
|  | Flonicamid | 7,158 | 0.056 | (0.006–0.867) |  | | 1,659 | | 0.072 | (0.006–0.824) |  | | 0.011 | |  |
|  | Imidacloprid | 7,158 | 0.016 | (0.007–0.149) |  | | 1,659 | | 0.016 | (0.007–0.142) |  | | 0.005 | |  |
|  | Imidacloprid-olefin | 7,158 | 0.234 | (0.127–1.011) |  | | 1,659 | | 0.234 | (0.127–1.011) |  | | 0.007 | |  |
|  | Nitenpyram | 7,158 | 0.014 | (0.009–0.154) |  | | 1,659 | | 0.014 | (0.009–0.128) |  | | 0.016 | |  |
|  | Sulfoxaflor A | 7,158 | 0.001 | (0.0010–0.0014) |  | | 1,659 | | 0.001 | (0.0010–0.0014) |  | | 0.024 | |  |
|  | Sulfoxaflor B | 7,158 | 0.001 | (0.001–0.002) |  | | 1,659 | | 0.001 | (0.001–0.002) |  | | 0.014 | |  |
|  | Thiacloprid | 7,158 | 0.002 | (0.001–0.006) |  | | 1,659 | | 0.002 | (0.001–0.006) |  | | 0.021 | |  |
|  | Thiacloprid-amide | 7,158 | 0.004 | (0.003–0.004) |  | | 1,659 | | 0.004 | (0.003–0.004) |  | | 0.011 | |  |
|  | Thiamethoxam | 7,158 | 0.040 | (0.005–0.490) |  | | 1,659 | | 0.043 | (0.005–0.533) |  | | 0.036 | |  |
| Maternal urine (second/third trimester) | | | | | | | | | | | | | | | |
|  | Acetamiprid | 6,782 | 0.001 | (0.001–0.047) |  | | 1,534 | | 0.001 | (0.001–0.036) |  | | 0.008 | |  |
|  | Acetamipride-N-desmethyl | 6,782 | 0.262 | (0.020–3.227) |  | | 1,534 | | 0.223 | (0.020–2.668) |  | | 0.078 | |  |
|  | Clothianidin | 6,782 | 0.105 | (0.011–1.340) |  | | 1,534 | | 0.104 | (0.011–1.180) |  | | 0.016 | |  |
|  | Clothianidin-desmethyl | 6,782 | 0.073 | (0.047–0.367) |  | | 1,534 | | 0.073 | (0.047–0.339) |  | | 0.014 | |  |
|  | Dinotefuran | 6,782 | 0.162 | (0.010–5.797) |  | | 1,534 | | 0.149 | (0.007–5.080) |  | | 0.029 | |  |
|  | Flonicamid | 6,782 | 0.056 | (0.006–0.712 |  | | 1,534 | | 0.056 | (0.006–0.740) |  | | 0.008 | |  |
|  | Imidacloprid | 6,782 | 0.016 | (0.007–0.141) |  | | 1,534 | | 0.016 | (0.007–0.141) |  | | 0.030 | |  |
|  | Imidacloprid-olefin | 6,782 | 0.234 | (0.127–0.984) |  | | 1,534 | | 0.211 | (0.127–0.929) |  | | 0.040 | |  |
|  | Nitenpyram | 6,782 | 0.014 | (0.009–0.174) |  | | 1,534 | | 0.014 | (0.009–0.107) |  | | 0.043 | |  |
|  | Sulfoxaflor A | 6,782 | 0.001 | (0.0010–0.0014) |  | | 1,534 | | 0.001 | (0.0010–0.0014) |  | | 0.017 | |  |
|  | Sulfoxaflor B | 6,782 | 0.001 | (0.001–0.002) |  | | 1,534 | | 0.001 | (0.001–0.002) |  | | 0.009 | |  |
|  | Thiacloprid | 6,782 | 0.002 | (0.001–0.005) |  | | 1,534 | | 0.002 | (0.001–0.005) |  | | 0.047 | |  |
|  | Thiacloprid-amide | 6,782 | 0.004 | (0.003–0.004) |  | | 1,534 | | 0.004 | (0.003–0.004) |  | | 0.010 | |  |
|  | Thiamethoxam | 6,782 | 0.036 | (0.005–0.491) |  | | 1,534 | | 0.037 | (0.005–0.472) |  | | 0.008 | |  |
| SMD, standardized mean difference | | | | | | | | | | | | | | | |
